# The effect of different insertion techniques on the depth of cure and vickers surface micro-hardness of two bulk-fill resin composite materials

**DOI:** 10.4317/jced.53356

**Published:** 2017-02-01

**Authors:** Lamiaa-Mahmoud Moharam, Ahmed-Zohair El-Hoshy, Karim Abou-Elenein

**Affiliations:** 1 Restorative and Dental Materials Research department, National Research Centre, Giza, Egypt; 2 Operative Dentistry department, Faculty of Oral and Dental medicine, Cairo, Egypt

## Abstract

**Background:**

The aim of this study was to evaluate the Vickers surface micro-hardness and the depth of cure of two bulk-fill resin composites and one incremental-fill resin composite.

**Material and Methods:**

Two Bulk-fill dental resin composites (X-tra Fil, Voco; Sonic-FillTM 2, Kerr Corporation) and an incremental-fill dental resin composite (Filtek™ Z250 XT, 3M ESPE) were used. Sixty cylindrical specimens of 4 mm thickness were prepared using split Teflon moulds. Specimens were divided into six groups (n=10) according to the type of the material used and according to the insertion technique applied (bulk or incremental). Prepared specimens were stored dry in complete darkness at 37°C for 24 hours. All specimens were tested for their Vickers surface micro-hardness, on their top and bottom surfaces. The depth of cure of the tested specimens was assessed by calculating the hardness ratio for each specimen. The Vickers surface micro-hardness and depth of cure data were analyzed for normality using Kolmogorov-Smirnov and Shapiro-Wilk tests. Independent sample-t test was used to compare between two groups while One-way ANOVA was used to compare between more than two groups.

**Results:**

Significant difference in the Vickers surface micro-hardness and depth of cure values was demonstrated among the tested materials (*P*<0.0001). X-tra Fil recorded the highest mean Vickers micro-hardness value (94.05±1.05). Bulk-fill dental resin composites X-tra Fil and Sonic-Fill showed 0.980±0.005 and 0.921±0.020 depth of cure values (bottom/top hardness ratio) respectively while Z250 XT recorded 0.776±0.141.

**Conclusions:**

X-tra Fil showed highest Vickers surface micro-hardness values on both top and bottom surfaces, whether inserted in increments or bulk. Both bulk-fill resin composites showed higher depth of cure for both insertion techniques.

** Key words:**Depth of cure, Vickers surface micro-hardness, bulk-fill resin composite, insertion techniques.

## Introduction

Restoring prepared dental cavities with light-curing resin composites has been regarded as the gold standard. However, to apply and cure the resin composite in successive increments of limited thickness has shown to be time-consuming and increase the risk of incorporating oral fluids within the increments, (1) which adversely affects the mechanical and physical properties of the set material.

Recently, many dental practitioners prefer using more time-saving restorative procedures. Dental resin composite manufacturers, with a vision to simplify these procedures; have introduced a new category of dental resin composites, the so-called “bulk-fill” Materials, (2) that can be applied in a single increment (up to 4 mm), reducing the clinical steps. Bulk-fill resin composite materials have high color translucency, allowing the incident light to penetrate deeper into the resin composite (3). The innovative system of polymerization-initiation that led to shortening of light-curing time and increasing the depth of cure. Low polymerization shrinkage of these materials together with their high filler content, resulted in very low polymerization which, allowed for application of thicker resin composite layers (4). Surface hardness measurement has shown to be a practical method to indirectly determine the degree of monomer conversion for resin composite materials. Furthermore; hardness profiles can be used to alternatively measure the depth of cure of such resinous materials (5).

Therefore; the objective of this study was to gain more insight about bulk-fill resin composite materials by analyzing the effect of different insertion techniques on the Vickers surface micro-hardness and the depth of cure of two different bulk-fill dental resin composite materials.

## Material and Methods

This study has been approved by ethics committee. Two commercially available bulk-fill dental resin composites and one incremental-fill dental resin composite were used in the study. Materials’ description, manufacturers and composition were listed in  Table 1.

**Table 1:**
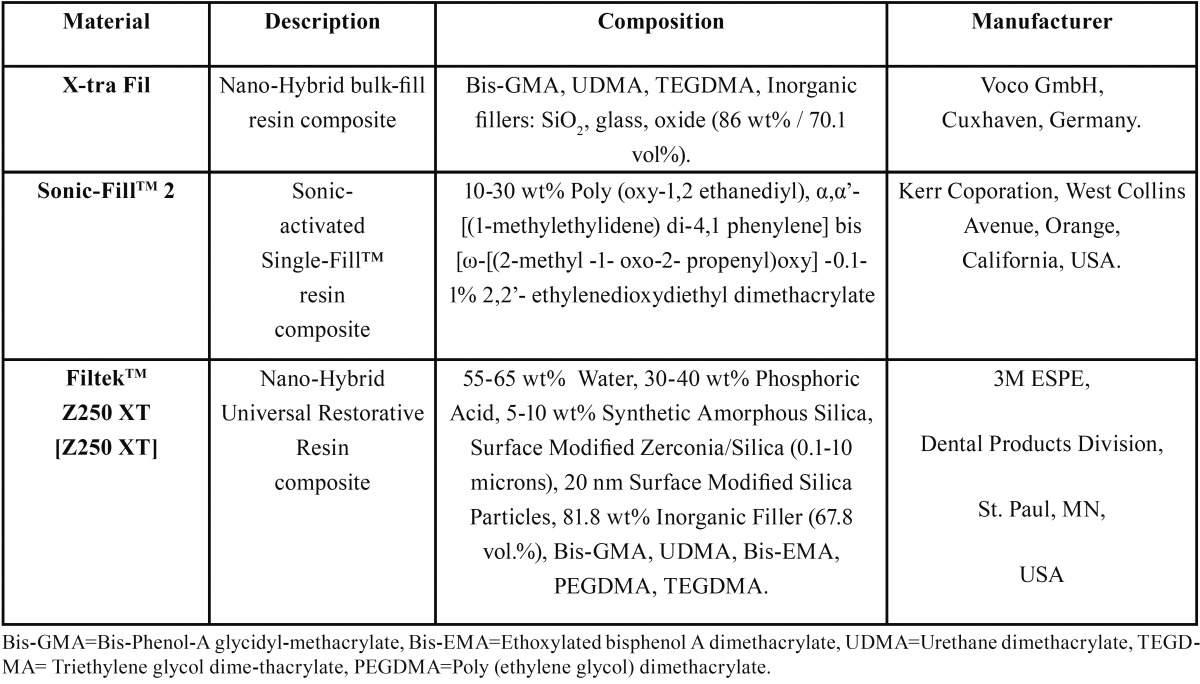
Materials description, composition and manufacturer.

-Study design and specimen grouping.

Sixty cylindrical specimens were prepared and assigned for the depth of cure and Vickers surface micro-hardness evaluation. Specimens were divided into six groups (n=10/group), representing the resin composite materials used in the study (two bulk-fill resin composites: Sonic-FillTM2 [Sonic-Fill] and X-tra Fil [X-tra Fil]); one incremental-fill resin composite: FiltekTM Z250 XT [Z250 XT]; and the two insertion techniques (bulk and incremental).

-Specimen preparation:

Split Teflon moulds of 4 mm diameter and 4 mm thickness were used to prepare the specimens. Each mould was encircled by a copper ring to provide stabilization during manipulation of the materials.

The moulds were first mounted on the top of a microscope slide and a clear Mylar strip, and then each mould was filled with one of the three resin composites (Sonic-Fill, X-tra Fil and Z250 XT) according to the insertion techniques investigated in the study. For the bulk insertion technique; each mould was filled in a 4 mm single increment. While for the incremental insertion technique; the resin composite materials were inserted into the moulds in two successive increments (2 mm thickness each).

The first resin composite increment was photo-polymerized using LED light curing unit (Elipar S10, 3M ESPE; USA) with a light intensity output of 1000 mW/cm2 for 20 seconds as recommended by the manufacturers of the control resin composite material used in the study (Filtek Z250 XT nano-hybrid universal incremental-fill resin composite); then the second resin composite increment was inserted and packed over the previously photo-polymerized increment till the mould was slightly over-packed. The top surface of each mould was then covered using a second clear Mylar strip to avoid creation of the oxygen inhibited layer. A glass slide and a load of 1 kg was constantly held in place on the top of the second clear Mylar strip for 30 seconds to ensure consistent packing of the specimens and to obtain flat surface (6).

The top surface of each prepared specimen was photo-polymerized using the light curing unit for 20 seconds. The guidance tip of the light curing unit was centered in 90° angle to the top surface of the specimen and kept in a direct contact with the second Mylar strip. Light intensity output of the light curing unit was monitored using Demetron radiometer device [Model 100, Demetron Research Corporation, Danbury, CT, USA] (7) and then the cylindrical specimens were gently removed from the moulds and the excess resin composite material was removed using a sharp scalpel.

Top surface of each specimen was identified with a dark marker pen. Specimens were stored dry in tightly sealed containers and in complete darkness condition at 37°C for 24 hours to prevent the ambient light from causing post light-curing polymerization, (7) until the Vickers micro-hardness test was conducted and the depth of cure was calculated.

-Vickers surface micro-hardness testing:

Sixty specimens (n=10) were tested using Vickers micro-hardness testing machine (Nexsus 4503, INNOVATEST, Netherlands, Europe) (7). Three randomized indentations on the center of the top and the bottom surfaces of each specimen were made using a diamond, square-head indentor at 500 g load and 15 seconds dwell time. Calculations were made using computer software (Hardness-Course Vickers/ Brinell/ Rockwell copy right IBS 2012 version 10.4.4) (8).

-Depth of cure of the resin composites:

The depth of cure of the tested resin composites was assessed by calculating the hardness ratio for each specimen. It was calculated by dividing the mean Vickers hardness number (VHN) of the bottom surface by the mean (VHN) of the top surface for each specimen according to the equation: (9) Depth of cure (hardness ratio) = VHN bottom surface / VHN top surface.

-Statistical analysis

Mean and standard deviation values were calculated for each group in each test. Data were explored for normality using Kolmogorov-Smirnov and Shapiro-Wilk tests. Independent sample-t test was used to compare between two groups while One-way ANOVA was used to compare between more than two groups. The significance level was set at *P*≤0.05. Statistical analysis was performed with IBM® SPSS® Statistics Version 20 for Windows.

## Results

Means and standard deviations of Vickers surface micro-hardness for the evaluated groups were represented in  Table 2. Significant difference was demonstrated between the mean micro-hardness values of different groups at *P*=0.001. The top surface group of X-tra Fil bulk-fill resin composite when used in bulk insertion technique recorded the highest mean micro-hardness value (95.16±0.44). However, the bottom surface group of Z250 XT recorded the lowest mean micro-hardness value when used in bulk insertion technique (57.16±0.69).

**Table 2:**
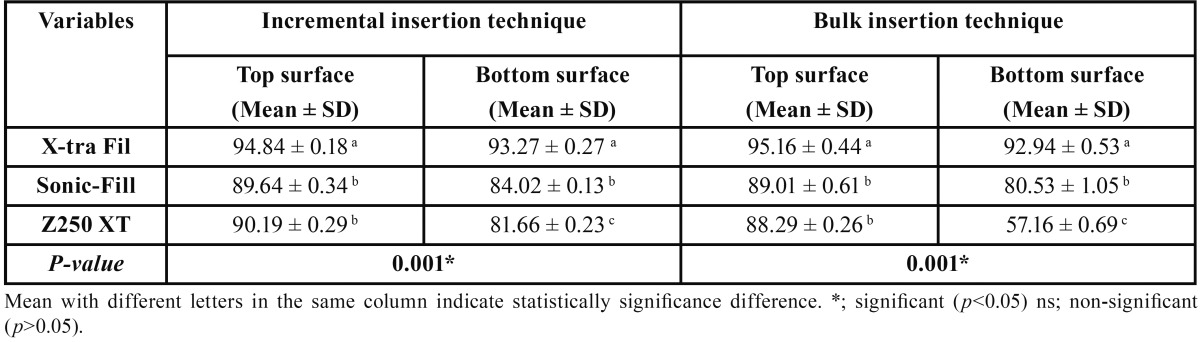
One-way ANOVA representing the mean, standard deviation (SD) values of Vickers surface micro-hardness of the different tested groups.

Mean and standard deviation values of the depth of cure of the tested materials were represented in  Table 3. A statistically significant difference was demonstrated between the depth of cure values of different groups at *p*=0.0001. The highest mean depth of cure value was recorded for X-tra Fil bulk-fill resin composite when inserted incrementally (0.983±0.001); while the lowest value was recorded for Z250 XT resin composite when inserted in bulk.

**Table 3:**
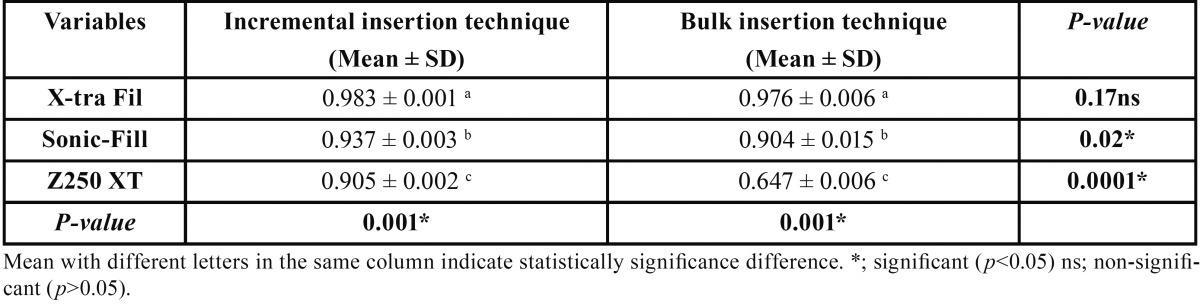
One-way ANOVA for the mean, standard deviation (SD) values of depth of cure of the different resin composite tested materials.

## Discussion

In the present study, the highest mean surface micro-hardness value was recorded for the X-tra Fil bulk-fill resin composite material. Statistically significant differences were noticed in Vickers surface micro-hardness mean values among all tested resin composite materials and that was in agreement with the previous findings of Leprince *et al.* (10).

Such findings could be multi-factorial. One of these factors might be the difference in the chemical composition of the resin monomer, which was reported to affect the surface micro-hardness of the resin composites (7).

It was stated that, mass fractions, size as well as distribution of filler particles within the resin monomer have a significant positive effect on the different physical and mechanical properties of the resin composites, including surface hardness, depth of cure, the degree of conversion, flexure and compressive strength (11). Manufacturers of bulk-fill dental resin composites have clarified that these materials have higher filler particles content and thus have increased esthetic, physical and mechanical properties (12).

In the current study; different resin composite materials were applied in increments (single or multiple) and then each increment was photo-polymerized only from the topside in a way to mimic the actual clinical situation. This would eventually mean less resin composite matrix polymerization and, accordingly, a larger role of the filler particle type and percentage (content) in the material behavior (13).

It is expected for any resin composite material that its top surface micro-hardness value to differ from the values recorded from the bottom surface, due to the difference in the monomer reactivity and filler/matrix refractive index mismatch (14).

In the present study, the mean micro-hardness values recorded on the bottom surfaces of Z250 XT were significantly decreased compared to the values recorded on the top surface; however, for X-tra Fil and Sonic-Fill bulk-fill resin composite materials; the values of the micro-hardness of the bottom surfaces were not statistically different from values registered for the top surfaces. The surface micro-hardness of resin composites was proved to be affected by increment thickness of the used resin composite material (7). In our study, two different insertion techniques of the resin composite materials were employed; which was expected to have the same effect on the surface micro-hardness at different specimen thickness. There was a decrease in the micro-hardness values on the bottom surfaces for all resin composite tested materials when bulk insertion technique was used. However; such change was only statistically significant for Z250 XT resin composite material.

The significant decrease in the surface micro-hardness of the 4 mm bulk inserted specimens of Z250 XT in this study were in agreement with a previous study (7) which reported that the resin Vickers hardness at the bottom surface was significantly different from that at the top surface when the specimens were placed in 4 mm-thick increments. Such finding may be explained by the difference in translucency between the bulk-fill and the incrementally inserted dental resin composites. With a higher translucency, the bulk-fill resin composites might have allowed more of the photo-polymerizing light to penetrate deep inside the resin composite materials, which possibly could have caused more polymerization of the resin composites monomers (15). This was also confirmed by previous studies reporting that enhanced polymerization of bulk-fill composite is owed to their higher translucency due to the increase in their filler particle size (7).

The significant difference between the surface micro-hardness values of X-tra Fil and Sonic-Fill bulk-fill resin composite materials might be attributed to the difference in their monomer viscosity. It was reported that the flowable bulk fill resin composites showed lower surface micro-hardness values than the condensable bulk-fill resin composites (16). Sonic-Fill resin composite material incorporates a highly filled resin monomer with special modifiers that strongly react to the sonic energy. Whenever the sonic energy is applied, the modifier causes the monomer viscosity to drop (up to 87%), increasing the flowability of the resin composite. Such drop might be responsible for the lower surface micro-hardness value (17).

Moreover, there are other parameters that might be responsible for the difference of surface micro-hardness values among the different tested materials including, filler particles morphology and distribution, (7) particle shape and density, monomer type and ratio, the degree of polymers cross-linking as well as the degree of conversion; which all vary greatly between the different products present in the market. A different study (18) showed that a ratio of 80% depth of cure has often been used as the minimum clinically acceptable value. In agreement with the results of other investigation (19) the hardness ratio of all materials tested in this study succeeded to fulfill this minimum value.

On the contrary, Z250 XT failed to fulfill this requirement when inserted in bulk. The higher depth of cure in bulk-fill resin composites could be owed to their higher translucency, as well as to their modified resin monomers and the recent photo-initiator systems (7,15). It was reported that the translucency of resin composites depends on their thickness as well as the scattering and absorption coefficients of the resin filler particles (7,13).

Some studies have shown that the translucency was decreased when the amount of the reinforcing filler particles was increased, (20) while it showed an increases when the reinforcing filler particles size was increased. This could be due to light scattering that happens within the resin composite matrix which might increase as the particle size of the filler approaches the wavelength of the activating light. It is well known that smaller filler particles scatter more light than larger filler particles (21). Such light scattering decreases the amount of the incident light which is transmitted throughout the resin composite and however interferes in a negative pattern with the physical and mechanical properties of the resin composites (22).

This was confirmed by results of the current study where Z250 XT showed significantly lower depth of cure than X-tra Fil and Sonic-Fill. There is some other factor that might explain the differences in the depth of cure among the different tested resin composite materials; which is the resin monomer properties. Moreover; the viscosity of the resin monomer and the flexibility of its compositional chemical structure might influence the depth of cure of the resin composite material. In the current study, X-tra Fil showed significant higher depth of cure than that of the other tested materials. This might be due to the difference in the chemical composition of their organic resin matrix. The organic resin matrix of X-tra Fil is composed of Bis-GMA, UDMA, and TEGDMA. It was stated that the ultimate degree of monomer conversion of the different resin composite systems, regarding their compositional resin monomers, increases in the subsequent arrangement: Bis-GMA<Bis-EMA<UDMA<TEGDMA (23).

Bis-GMA has a strong intra-molecular hydrogen bonding of its hydroxyl groups, in order that it is considered the most viscous and least flexible monomer among the other compositional dental resin monomers. UDMA has a hydrogen bond between its amine and carbonyl groups, and it is also a viscous resin monomer.

Nevertheless, the viscosity of UDMA is much lower in relation to that of Bis-GMA due to its weaker hydrogen bond.

Additionally, amine groups presence in the urethane structure of UDMA monomer is considered to be responsible for the distinguishing chain transfer reactions that offer an alternative trail for the continuance of polymerization; therefore resulting in a promoted resin monomer conversion (24). This might explain the higher degree of conversion and depth of cure values of UDMA containing organic matrix of X-tra Fil. Although, Z250 XT organic matrix is similar to that of X-tra Fil but it recorded lower depth of cure. This is possibly due to the polymerization characteristics, which were significantly influenced by the difference in organic resin matrix chemistry and by the concentration of each resin monomer in the composite matrix (25). TEGDMA is considered to be a diluent monomer, as it has the lowest viscosity and the highest degree of monomer conversion among the different resin composite monomer systems. Therefore, when Bis-GMA is diluted with the low viscosity TEGDMA resin monomer, a synergistic effect has been significantly observed on the rate of polymerization, degree of monomer conversion as well as the depth of cure (26). Consequently, this might be another helpful factor that might explain the significant high depth of cure of the X-tra Fil bulk-fill resin composite material since it may contain a higher concentration of TEGDMA monomer of resin composite.

When incremental insertion was used, a significantly higher depth of cure was noted compared to bulk insertion. Such results were confirmed by a previous study and might be related to the fact that specimens prepared with incremental packing received more total energy than those inserted in bulk (27).

## Conclusions

X-tra Fil bulk-fill resin composite material has showed the highest Vickers surface micro-harness values on both top and bottom surfaces of the tested specimens, whether inserted in increments or in bulk. Both bulk-fill resin composites (X-tra Fil and Sonic-Fill) showed higher depth of cure regarding both insertion techniques.
